# Convalescent Plasma Reduces Mortality and Decreases Hospitalization Stay in Patients with Moderate COVID-19 Pneumonia

**DOI:** 10.3390/metabo11110761

**Published:** 2021-11-05

**Authors:** Maamoun Basheer, Elias Saad, Dorin Shlezinger, Nimer Assy

**Affiliations:** 1 Internal Medicine Department, Galilee Medical Center, Nahariya 2210001, Israel; maamon.basheer@mail.huji.ac.il (M.B.); eliass@gmc.gov.il (E.S.); dorinshl307@gmail.com (D.S.); 2 The Azrieli Faculty of Medicine, Bar-Ilan University, Safad 2210001, Israel

**Keywords:** convalescent plasma therapy, mortality, moderate COVID-19 patients, time to discharge, hospitalization length, inflammatory markers

## Abstract

Humans infected with SARS-CoV-2 may develop COVID-19, which manifests across a wide spectrum of clinical severity ranging from mild upper respiratory tract illnesses to diffuse viral pneumonia, causing acute respiratory failure. Many therapies have been tested for their efficacy in treating COVID-19. Controversy surrounds convalescent plasma transfusions as an effective treatment for COVID-19. This study discusses the efficacy of this treatment on COVID-19 patients. Electronic medical record data were collected from patients diagnosed with COVID-19, from November 2020 to August 2021, in the Galilee Medical Center’s COVID-19 departments. Epidemiological, clinical, laboratory and imaging variables were analyzed. Multivariate stepwise regression and discriminant analyses were used to identify and validate the correlation between convalescent treatment and either death or time to negative PCR and hospitalization length. The study population included 270 patients, 100 of them treated with convalescent plasma. The results show that convalescent plasma therapy significantly prevented mortality in moderate patients, reduced hospitalization length and time to negative PCR. Additionally, high BMI, elderly age, high CRP and 4C-scores correlated with the severity and mortality of COVID-19 patients. Convalescent plasma also significantly reduced inflammatory markers, especially in moderate COVID-19 patients. In non-critical hospitalized patients, convalescent plasma therapy reduces morbidity and mortality in moderate COVID-19 patients and hospitalization length. Identifying patients who could benefit from this treatment could reduce the risk of death and shorten their hospitalization stay.

## 1. Introduction

SARS-CoV-2 infection can develop into COVID-19, which manifests across a wide spectrum of clinical severity, ranging from a mild upper respiratory tract illness to diffuse viral pneumonia causing acute respiratory failure, with sequelae including acute lung injury, multiorgan dysfunction syndrome and death [1,2,3].

The virus itself causes lymphocyte apoptosis, impairing their function, and ending in a fulminant cytokine storm [4]. This condition resembles secondary hemophagocytic lymphohistiocytosis or macrophage activation syndrome and can progress to acute respiratory distress syndrome (ARDS) and multiorgan failure [5].

Patients with a mild clinical presentation may not initially require hospitalization. This decision will depend on the clinical presentation, requirement for supportive care, potential risk factors for severe disease, and the patient’s ability to self-isolate at home [6]. Patients with severe presentations require hospitalization. In-patient management revolves around the supportive management of the most common complications of severe COVID-19, like pneumonia, hypoxemic respiratory failure, ARDS, sepsis and septic shock, acute kidney injury, along with complications from prolonged hospitalization [7].

Several therapeutic agents have been evaluated for treating COVID-19 like the antiviral drug Remdesivir, convalescent plasma and dexamethasone [7]. The Food and Drug Administration issued an emergency use authorization for COVID-19 convalescent plasma for treating hospitalized patients with COVID-19 [6]. Although protection from COVID-19 infection or disease has yet to be directly correlated with levels of circulating antibodies against SARS-CoV-2 [8], providing virus-neutralizing antibodies in the form of convalescent plasma may expedite disease resolution before the maturation of the patient’s own humoral response [8,9]. There is currently insufficient data to recommend either for or against the use of convalescent plasma for the treatment of COVID-19 [10,11,12]. In respiratory infections specifically, the strongest evidence suggests that the benefit of passive antibody transfer is most demonstrable in patients who were treated within days of symptoms onset [13,14]. 

This study proposes to identify the efficiency of convalescent plasma as a treatment for COVID-19 patients. The end parameters are death, hospitalization stay and time to negative PCR. This study also assesses the correlation between disease severity, presented as blood inflammatory marker levels and the reduction of these markers after plasma transfusion.

## 2. Results

### 2.1. Clinical Characteristics of the Patients Treated with or without Convalescent Plasma

The data of the 270 COVID-19 patients who fit the inclusion criteria were analyzed. Of these patients, 100 patients were treated with convalescent plasma, and 170 patients were not treated with convalescent plasma. There were no significant differences between the two groups in the demographics, symptoms and laboratory findings upon admission [1]. All patients in both groups were treated with corticosteroids (Table 1). There was a difference in genders with the plasma-treated group having significantly more males than in the untreated group (61 vs. 51, respectively) (Table 1). 

The untreated group had more use of high flow (30% vs. 10%, respectively) than in the convalescent plasma-treated group. A significant reduction in time to discharge (in days) was seen in the treated group compared to the untreated group (13 ± 3.6 vs. 12.4 ± 5.2, respectively) and also time to negative PCR (13 ± 3.6 vs. 15.1 ± 11.3, respectively) 

### 2.2. Association between Clinical Parameters, Convalscment Plasma Therapy and Mortality in COVID-19 Patients 

Many parameters, including convalescent plasma therapy, age, BMI, ALT, HDL, BUN, CRP, Ferritin, NLR, 4C-score and SOFA score were studied for their correlation to mortality in the all severity COVID-19 patients’ groups. The results show that convalescent plasma therapy significantly prevented mortality (Table 2) in non-hypoxemic moderate patients. Correlation of all patient severity groups who were treated with convalescent plasma with patients who were not treated with convalescent plasma did not show any difference in mortality (Table 2). Additionally, high BMI, elderly age, high CRP and a 4C-score significantly correlated with mortality (Table 2) in all severity groups.

The Plasma was given within 8 days after the first symptoms. There was no correlation between plasma treatment, symptom duration and death.

### 2.3. The Effect of Convalescent Plasma on the Hospitalization Length and Time to Negative PCR 

**Figure 1:**
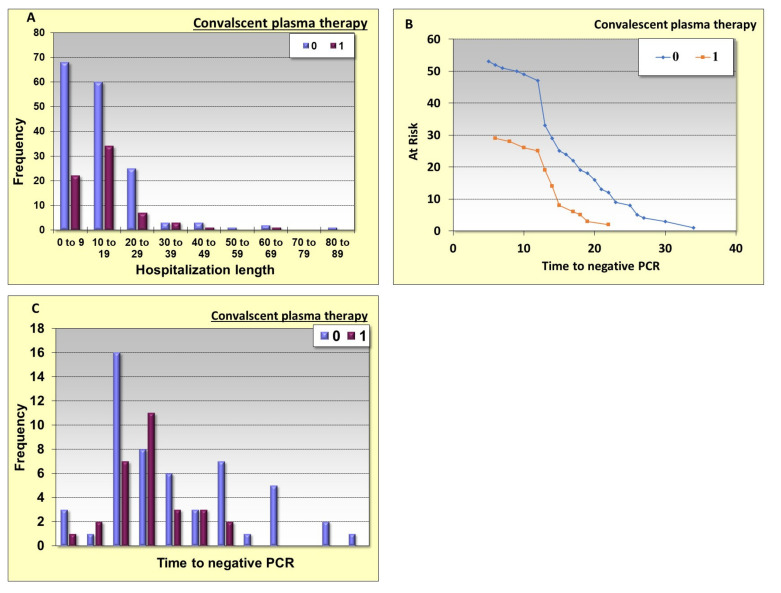
The effect of convalescent plasma on hospitalization length and time to negative PCR. (**A**): The frequency of patients in different categories of hospitalization length. (**B**,**C**): Correlations between convalescent plasma and time to negative PCR. The 0,1 (blue and red columns) are the patients who were not treated with convalescent plasma and those treated with it, respectively. The time measured in days of hospitalizations.

Clinical parameters, laboratory parameters and convalescent plasma therapy were analyzed for their ability to affect hospitalization length and time to negative PCR. Convalescent plasma therapy significantly correlates with short hospitalization length and reduced time to negative PCR (Table 3A,B). Correlations of all patient severity groups who were treated with convalescent plasma with patients who were not treated with convalescent plasma did not show any difference in hospitalization length and time to negative PCR (Table 3A,B). Low 4 C-score and low NLR correlated significantly with short hospitalization stay and reduced time to negative PCR in all patients’ severity groups, respectively (Table 3A,B). The frequency of patients in different categories of hospitalization length can be seen in Figure 1A. Patients who were treated with convalescent plasma show shorter hospitalization lengths. Convalescent plasma therapy is correlated with short time to negative PCR, while high NLR is not (Figure 1B,C).

### 2.4. The Effect of the Convalescent Plasma on COVID-19 Patients’ Inflammatory Status 

**Figure 2:**
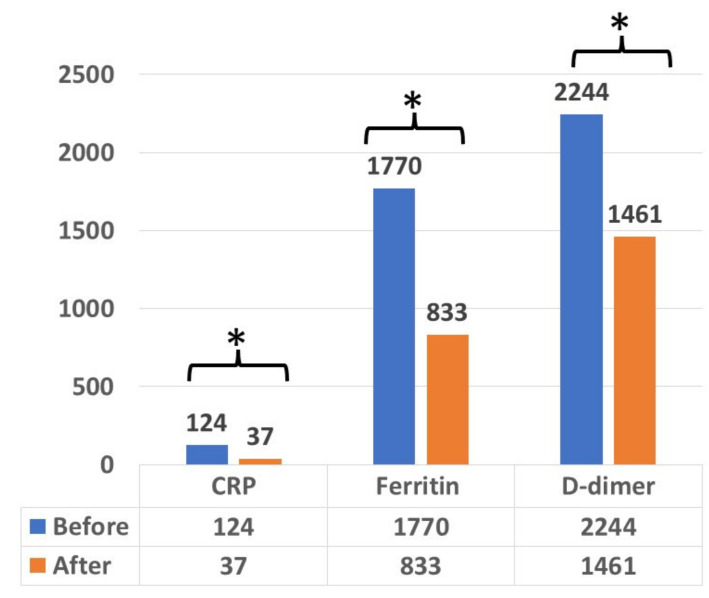
The effect of convalescent plasma on inflammatory markers (D-dimer, ferritin and CRP) in all patients treated with convalescent plasma. The measurements were done upon admission and 3–5 days after convalescent plasma perfusion. The asterisks mean the observed significance levels. (*p*-value < 0.05).

To identify the effect of convalescent therapy on inflammatory status, measurements of the cytokines and inflammatory markers were done upon admission and 3–5 days after convalescent plasma perfusion. Convalescent plasma reduced the inflammatory markers (D-dimer, ferritin and CRP) after admission, as seen in Figure 2. 

## 3. Discussion

This study probed the efficiency of convalescent plasma as a treatment for different severities of COVID-19 patients. The results show that convalescent plasma therapy significantly prevents mortality in moderate patients, reduced hospitalization length and also time to negative PCR. Convalescent plasma also significantly reduced inflammatory markers, especially in moderate COVID-19 patients. We also found that high BMI, elderly age, high CRP and 4C-scores correlated with the severity and mortality of COVID-19 patients. 

The use of antibody-rich plasma is not new. Patients in past pandemics received influenza-convalescent human blood products and somewhat clinically benefited from them [15]. Convalescent plasma or immunoglobulins have been used to improve the survival rate of patients with SARS, whose condition continued to deteriorate despite treatment with pulsed methylprednisolone [16]. Moreover, several studies show a shorter hospital stay and lower mortality in COVID-19 patients treated with convalescent plasma than those who were not treated with convalescent plasma [16,17,18]. However, some researchers show that in patients with severe, or life-threatening COVID-19, convalescent plasma therapy added to standard treatment, compared with standard treatment alone, did not result in a statistically significant improvement in time to clinical improvement within 28 days [19]. Our data also confirm that convalescent plasma is not clinically beneficial in severe COVID-19 patients but only in moderate patients. Convalescent plasma is an anti-SARS-CoV-2 rich medium and is a suitable treatment for the viral phase of the Coronavirus infection, before deteriorating to the inflammatory phase. Thus, convalescent plasma therapy helps patients who present to the emergency room within a few days after Coronavirus infection. These antibodies neutralize the viruses in the early phase but do not affect the virus in the late phases, like secondary hemophagocytic lymphohistiocytosis or macrophage activation syndrome [5]. We also should emphasize that all patients in both groups were treated with corticosteroids. So convalescent plasma is an additional factor. Although recent research showed that in patients hospitalized with COVID-19, the use of dexamethasone resulted in lower 28-day mortality among those who were receiving either invasive mechanical ventilation or oxygen alone at randomization but not among those receiving no respiratory support [20] 

The possible mechanisms of action of convalescent plasma include direct neutralization of the virus, suppression of an overactive immune system and immunomodulation of the hypercoagulable state [21,22]. The antibodies mediate inhibitory effects on the virus’s cell entry ability, thus limiting their amplification. These antibodies also activate other pathways, such as the complement cascade, antibody-dependent cellular cytotoxicity and phagocytosis [21,22]. This could help explain how plasma helps the immune system to eradicate the virus. This also explains our results about the short time to negative PCR and a short stay in the convalescent plasma therapy group. This effect is also better achieved if used in non-critical hospitalized patients. 

Some studies have shown that IgG transferred by plasma neutralizes cytokines, such as IL-1β and TNFα [23,24]. Therefore, passive immunity by infusing convalescent plasma may limit the inflammatory cascade driven by pathogenic antibodies, as well as the cellular damage induced by the complement cascade activation [23,24,25,26]. Our results show that the use of convalescent plasma as a therapy to non-critical COVID-19 patients significantly improved their inflammatory status, especially the D-dimer, CRP and ferritin markers.

This study emphasizes that high BMI, elderly age, high CRP and 4C-scores correlate with the severity and mortality of COVID-19 patients. These results fit our recent study which showed four predictive factors associated with increased disease severity and mortality in COVID-19 patients [27]. 

Our study has some limitations. The supportive data presented here were largely obtained from reports that emerged early during the COVID-19 pandemic. In addition, the wide diversity of study methodologies, statistical approaches, modest sample sizes and geographic sites may have confounded our interpretation of the data. Our study is a retrospective study with all its limitations. The retrospective aspect may introduce selection bias and misclassification.

## 4. Conclusions

This study identifies the efficiency of convalescent plasma as a treatment for different severities of COVID-19 patients. The results show that convalescent plasma therapy significantly prevents mortality in moderate patients, reduces hospitalization length and time to negative PCR. Identifying patients who could benefit from this treatment could reduce the risk of death and shorten their hospitalization stays.

## 5. Methods 

### 5.1. Study Population

Electronic medical records (EMR) were analyzed from patients diagnosed with COVID-19 from November 2020 to August 2021, at the Galilee Medical Center COVID-19 Department, Nahariya, Israel. The infection was based on a positive polymerase chain reaction (PCR). EMR data from 270 patients were analyzed. Of them, 100 were treated with convalescent plasma and the other 170 patients were not.

### 5.2. Study Design

The analysis was done on Electronic medical records from the COVID-19 patients. The symptoms duration before admission (fever, myalgia, dyspnea and diarrhea), demographic background, past medical history and treatments, weight, BMI, time to discharge or time to death, time to negative nasopharynges RT-PCR test and treatments upon hospitalization, blood laboratory tests (biochemistry, CBC, blood gases, blood type, coagulation tests and inflammatory markers), the confirmation of SARS-CoV-2 infection was done by positive real-time PCR assays from nasal and nasopharyngeal samples. Scores like the 4-C score and SOFA were calculated. The 4-C score was analyzed as described by Knight et al. [28].

### 5.3. Eligibility Criteria

#### 5.3.1. Inclusion Criteria

(1) Adult males or females ≥18 to ≤80 years of age
(2) Proven COVID-19 infection per RT-PCR assay of a pharyngeal sample (nasopharyngeal or oropharyngeal)
(3) Severe or moderate (patient without hypoxemia) patients as defined by the Israeli Ministry of Health.

Severe patients were defined as having two of the following criteria:

- Pneumonia, defined as radiographic opacities on the chest X-ray or CT scan
- Saturation below 94% at room air
- PAO_2_/FiO_2_ < 300

#### 5.3.2. Exclusion Criteria

(1) Pregnant (positive serum or urine test within 3 days prior to randomization)
(2) Confirmed infection of more than 10 days
(3) Need for chronic oxygen use
(4) Confirmed bacteremia
(5) Life expectance below 48 hours
(6) Failure of three systems
(7) Age >80 to <18

### 5.4. Severity of Illness Categories

Asymptomatic or pre-symptomatic infection: Individuals who test positive for SARS-CoV-2 but have no symptoms.

Mild illness: individuals who in addition have any of the various signs and symptoms of COVID-19 (e.g., fever, cough, sore throat, malaise, headache and muscle pain) without dyspnea or abnormal chest imaging.

Moderate illness: Individuals who have evidence of lower respiratory disease by clinical assessment or imaging and oxygen saturation is equal or above 93% in room air. 

Severe illness: Individuals who have respiratory frequency above 30 breaths per minute, Spo2 < 94% in room air or PaO2/FiO2 < 300 mmHg. 

Critical illness: Individuals who have respiratory failure, septic shock, or multiple organ dysfunctions. 

Discontinuation Criteria: Patients who developed transfusion-related acute lung injury (TRALI) or respiratory distress during plasma perfusion.

### 5.5. Ethics

Approval of the study by our medical center’s local ethics committee was done. The study approval number is (0244-20-NHR). The study was performed under the oversight of the ICH guidelines for good clinical practice.

### 5.6. Statistical Analysis

WinSTAT program was used to resume the statistical analysis. Results were presented as mean + SD. The frequency and corresponding diagnosis percentage were provided. Clinical and biochemical variables as independent variables were analyzed, the univariate direct regression analysis and multivariate stepwise regression analysis were performed. Survival or death are the dependent variables. The significance level was less than 0.05. 

## Figures and Tables

**Table 1:** Clinical and laboratory characteristics of the COVID-19 patients who were treated with or without convalescent plasma (*p*-value < 0.05).

Variable	with Convalescent Plasma	without Convalescent Plasma	*p*-Value
Total	n = 100	n = 170	
Age	62 ± 14	64 ± 17	0.4
Male (%)	67%	51%	0.005
BMI	30 ± 5	29 ± 5.5	0.3
**Comorbidities %**			
Diabetes (%)	42	38	0.5
Hypertension (%)	58	58	0.5
Lung disease (%)	14	8	0.3
Hemodialysis (%)	6	6	0.2
Aspirin use (%)	35	40	0.3
**Symptom’s duration before admission to hospitals** **(days)**	6 ± 5	8 ± 1.2	0.19
**Symptoms before admission** **(% of total)**			
Fever %	69	62	0.77
Diarrhea %	8	14	0.26
Dyspnea %	55	46	0.74
Clinical severity on admission %	57	57.6	0.5
**Lab Findings upon admission**			
Hemoglobin (mg/dL)	12.7 ± 2	11.8 ± 1.8	0.14
Absolute neutrophil count (×10^3^/µL)	6 ± 3	7.2 ± 6.1	0.9
Absolute lymphocyte count (×10^3^/µL)	1.2 ± 1.1	1.5 ± 3	0.5
Neutrophil to lymphocyte ratio (NLR)	8.3 ± 7.8	8.9 ± 3.9	0.8
Platelet (×10^3^/µL)	212 ± 84	224 ± 9	0.79
BUN (mg/dL)	25 ± 20	24.3 ± 19	0.6
Creatinine (mg/dL)	1.3 ± 1.6	1.7 ± 3	0.7
Triglycerides (mg/dL)	166 ± 14	170 ± 24	0. 8
HDL (mg/dL)	26 ± 9	32.6 ± 11	0.001
C-reactive protein (CRP) (mg/dL)	124 ± 86	109 ± 89	0.1
Ferritin	1020 ± 387	1043 ± 325	0.57
D-dimer	1426 ± 117.2	1576 ± 209.8	0.5
Fibrinogen	739 ± 146	704 ± 199	0.33
IL-6	82 ± 75	68 ± 10	0.2
ALT	38 ± 36	40 ± 69	0.1
4-C score	9 ± 1	8.8 ± 7.6	0.7
SOFA score	3 ± 1.8	2.3 ± 1.7	0.01
O_2_ supplement on admission %	57	57	0.5
High flow use (% of total)	10	30	0.001
Hospitalization length (days)	12.4 ± 5.2	13.2 ± 8.9	0.001
Time to negative PCR (days)	13 ± 3.6	15.1 ± 11.3	0.05
**Treatment in hospitalization**			
**Convalescent plasma therapy**	100	0	0.001
LMWH (% of total)	100	100	
Vitamin D (% of total)	100	100	
Corticostriods (% of total)	100	100	

**Table 2:** Correlations between risk factors and mortality in COVID-19 patients. Univariate analysis of the strength of risk factors with mortality prediction. Convalescent plasma prevents mortality in moderate patients. Abbreviation: BMI body mass index: SE are the standard errors of the regression coefficients. T is the quotient of the coefficient. Two-sided *p* values or observed significance levels.

	Coefficient	*p*-Value	T	Std. Error	95% Conf. (±)
Convalescent plasma therapy in moderate patients	−0.7	0.0001	−4.2	0.1	0.33
Convalescent plasma all patients	0.05	15	0.8	0.5	0.09
Age	0.01	0.001	+3.3	0.003	0.01
BMI	0.01	0.02	+2.2	0.008	0.01
CRP	0.001	0.02	+2.3	0.0005	0.001

**Table 3:** The effect of the convalescent plasma on the hospitalization length (**A**) and the time to negative PCR (**B**) in moderate patients. SE are the standard errors of the regression coefficients. T is the quotient of the coefficient. Two-sided *p* values or observed significance levels.

A					
	Coefficient	95% Conf. (±)	Std. Error	T	*p*-Value
Constant					
**Convalescent plasma therapy in moderate patients**	**2.5**	**2.05**	**1.0**	**2.4**	**0.02**
**Convalescent plasma therapy in all patients**	**4.8**	**5.7**	**2.9**	**1.67**	**0.1**
**4C-Score**	**0.77**	**0.52**	**0.25**	**3.0**	**0.005**
**B**					
	**Coefficient**	**95% Conf. (±)**	**Std. Error**	**T**	***p*-Value**
**Constant**					
**Convalescent plasma therapy**	**7.7**	**0.9**	**0.4**	**18.5**	**0.02**
**Convalescent plasma therapy in all patients**	**1.6**	**2.8**	**1.4**	**−1.1**	**0.3**
**NLR**	**−0.4**	**0.2**	**0.1**	**−3.4**	**0.005**

## Data Availability

The data presented in this study are available on request from the corresponding author. The data are not publicly available due to the participant consent obtained as part of the recruitment process, it is not possible to make these data publicly available.

## Abbreviation

EMR: electronic medical record. NLR: neutrophil to lymphocyte ratio, ARDS: acute respiratory distress syndrome, TRALI: transfusion-related acute lung injury
